# First Outbreak of an H5N8 Highly Pathogenic Avian Influenza Virus on a Chicken Farm in Japan in 2020

**DOI:** 10.3390/v13030489

**Published:** 2021-03-16

**Authors:** Saki Sakuma, Yuko Uchida, Momoyo Kajita, Taichiro Tanikawa, Junki Mine, Ryota Tsunekuni, Takehiko Saito

**Affiliations:** 1 Division of Transboundary Diseases, National Institute of Animal Health, National Agriculture and Food Research Organization (NARO), Ibaraki 3050856, Japan; sakumas438@affrc.go.jp (S.S.); ttanikawa@affrc.go.jp (T.T.); minejun84032@affrc.go.jp (J.M.); tune@affrc.go.jp (R.T.); taksaito@affrc.go.jp (T.S.); 2 Hokkaido Kamikawa Livestock Hygiene Service Center, Hokkaido 0718154, Japan; kajita.momoyo@pref.hokkaido.lg.jp

**Keywords:** H5N8, highly pathogenic avian influenza, Japan, 2020, chicken, animal experiment

## Abstract

On 5 November 2020, a confirmed outbreak due to an H5N8 highly pathogenic avian influenza virus (HPAIV) occurred at an egg-hen farm in Kagawa prefecture (western Japan). This virus, A/chicken/Kagawa/11C/2020 (Kagawa11C2020), was the first HPAI poultry isolate in Japan in 2020 and had multiple basic amino acids—a motif conferring high pathogenicity to chickens—at the hemagglutinin cleavage site. Mortality of chickens was 100% through intravenous inoculation tests performed according to World Organization for Animal Health criteria. Phylogenetic analysis showed that the hemagglutinin of Kagawa11C2020 belongs to clade 2.3.4.4B of the H5 Goose/Guangdong lineage and clusters with H5N8 HPAIVs isolated from wild bird feces collected in Hokkaido (Japan) and Korea in October 2020. These H5N8 HPAIVs are closely related to H5N8 HPAIVs isolated in European countries during the winter of 2019–2020. Intranasal inoculation of chickens with 10^6^ fifty-percent egg infectious doses of Kagawa11C2020 revealed that the 50% chicken lethal dose was 10^4.63^ and the mean time to death was 134.4 h. All infected chickens demonstrated viral shedding beginning on 2 dpi—before clinical signs were observed. These results suggest that affected chickens could transmit Kagawa11C2020 to surrounding chickens in the absence of clinical signs for several days before they died.

## 1. Introduction

Since 2003, highly pathogenic avian influenza viruses (HPAIVs) of the H5 subtype in which the *hemagglutinin* (*HA*) genes are derived from A/goose/Guangdong/1/1996 [1] —the so-called Goose/Guangdong (Gs/Gd) lineage—have been spreading to poultry and wild bird species worldwide, except in countries in South America and Oceania [2,3]. On 5 November 2020, an outbreak due to an H5N8 HPAIV was confirmed at an egg-hen farm where 300,000 of layer were raised in cages in Kagawa prefecture (western Japan). Eleven days before the outbreak (i.e., 24 October 2020), another H5N8 isolate, A/northern pintail/Hokkaido/M13/2020 [4], was detected in feces collected in Hokkaido prefecture (northern Japan). In addition, since the end of August 2020, H5N8 HPAIVs have been detected in wild bird and poultry species in Russia, Kazakhstan, Israel, European Union countries, and South Korea [3].

The first case of H5 HPAIVs of the Gs/Gd lineage in Japan was reported in 2004 [5]; the WHO/OIE/FAO H5N1 Evolution Working Group classified the *HA* gene of this isolate as belonging to clade 2.5 [6]. The HPAIVs of the Gs/Gd lineage reported in Japan in 2007, 2008, and 2010–2011 are H5N1 viruses in clades 2.2, 2.3.2, and 2.3.2.1, respectively [7,8,9]. Several times since 2014, Gs/Gd HPAIVs in clade 2.3.4.4B have been introduced into Japan [10,11,12,13]. The *neuraminidase* (*NA*) gene segment of these intruding clade 2.3.4.4B viruses was replaced from N1 to N8 during 2014–2015 [10,12] and from N1 to N6 during 2016–2017 [13] and 2018 [11].

In the current study, we characterized the H5N8 HPAIV A/chicken/Kagawa/11C/2020 (Kagawa11C2020: H5N8), isolated from the first H5N8 poultry case in Japan in 2020, by using genetic analysis and experiments in chickens.

## 2. Materials and Methods

### 2.1. Virus Isolation

A cloacal swab from a dead chicken at the affected farm was inoculated into embryonated eggs; infectious allantoic fluid was examined for hemagglutination activity at the Kagawa Municipal Animal Hygiene Center and then was submitted for further study at the National Institute of Animal Health of Japan.

### 2.2. Sequencing

Viral RNA was extracted by using an RNeasy Mini Kit (QIAGEN, Benelux B.V., Amsterdam, The Netherlands), followed by cDNA library preparation by the NEBNext Ultra II RNA Library Prep Kit for Illumina (NEB, Ipswich, MA, USA). The cDNA was sequenced with a Miseq sequencer by using Miseq Reagent Kit version 2 (lllumina, San Diego, CA, USA) as previously described [13]. Consensus sequences were generated by using FluGAS software (version 2.25; World Fusion, Tokyo, Japan).

### 2.3. Phylogenetic Analysis

Among all the H5 *HA* sequences downloaded from GISAID and NCBI on 10 December in 2020, the H5 *HA* genes of Gs/Gd isolates were extracted and subjected to CD-Hit analysis with a homology threshold of 99.5%, although the sequences of viruses closely related to Kagawa11C2020 were not reduced. The *HA* gene sequences of Kagawa11C2020 and closely related viruses were aligned by using MAFFT version 7 [14] and analyzed according to a generalized time-reversible (GTR) model by using Fasttree version 2.1.10 [15]. The phylogenetic tree was visualized by using FigTree (version 1.4.4, The University of Edinburgh, Edinburgh, UK) [16].

### 2.4. Animal Experiments

Chickens were inoculated with H5N8 HPAIVs in the Biosafety Level 3 facilities at the National Institute of Animal Health, Japan. Four or five chickens were kept in each isolator with not restricted food and water intake. Infectious allantoic fluid of Kagawa11C2020 contained 10^9.45^ EID_50/mL_ titer of the virus in each virus stock tube and was kept in −80 degree until used. The pathogenicity of the H5N8 Japanese strains, Kagawa11C2020, was evaluated by inoculation intravenously in 7-week-old chickens according to the Manual of Diagnostic Tests and Vaccines for Terrestrial Animals 2019 [17]. Eight of seven-week-old chickens (L-M-6 strain; Nisseiken Co., Ltd., Tokyo, Japan) were intravenously inoculated with 200 μL of a 1/10 dilution of the infectious allantoic fluid of Kagawa11C2020 containing 10^7.75^ EID_50_ titer of the virus in 200 μL. As a natural infection model, twenty of 4-week-old chickens were used for intranasal inoculation of Kagawa11C2020. One of four doses (10^2^ of fifty-percent egg infectious dose (EID_50_), 10^4^ EID_50_, 10^5^ EID_50_, and 10^6^ EID_50_) of virus was inoculated intranasally into five 4-week-old healthy White Leghorn chickens that had never been exposed to influenza virus(L-M-6 strain; Nisseiken Co., Ltd., Tokyo, Japan). Tracheal and cloacal swabs were collected when the chickens died or at 1, 2, 3, 5, 7, 10, and 14 days post-inoculation (dpi). The sample collection from chickens and viral titration of samples were conducted as previously described [18]. Influenza A Virus Antibody Test Kit (IDEXX Laboratories, Westbrook, ME, USA) was used to detect antibodies against type A influenza viruses in sera collected from surviving chickens at 14 dpi. Survival analysis according to log-rank testing was conducted by using SYSTAT 13.2 software (HULINKS, Tokyo, Japan) with Holm correction. The mean time to death (MTD) of infected chickens and the viral titers in tracheal and cloacal swabs obtained during animal experiments were compared between Kagawa11C2020 group and Japan isolate experimental groups by using Dunnett’s test.

## 3. Results and Discussion

The deduced amino-acid sequence of the *HA* cleavage site of Kagawa11C2020 is PLREKRRKR/GLFG, which includes the run of consecutive basic amino acids that is the characteristic motif of HPAIVs [19], suggesting that Kagawa11C2020 is highly pathogenic to chickens. High pathogenicity to chickens was confirmed through testing according to OIE criteria, because all of the seven-week-old chickens inoculated intravenously died within 48 h, resulting in a mortality rate of 100%.

Blast analysis to the GISAID database of each segment of Kagawa11C2020 revealed that they possessed high similarity to the viruses isolated in Europe from January to March, 2020 along with A/Mandarin duck/Korea/H242/2020 (GISAID isolate ID, EPI_ISL_631824) which was isolated in October 2020 in Korea. They were also highly homologous (99.0%; *NS* gene to 99.8%; *HA* gene) to A/northern pintail/Hokkaido/M13/2020 described by Isoda et al. [4].

**Figure 1:**
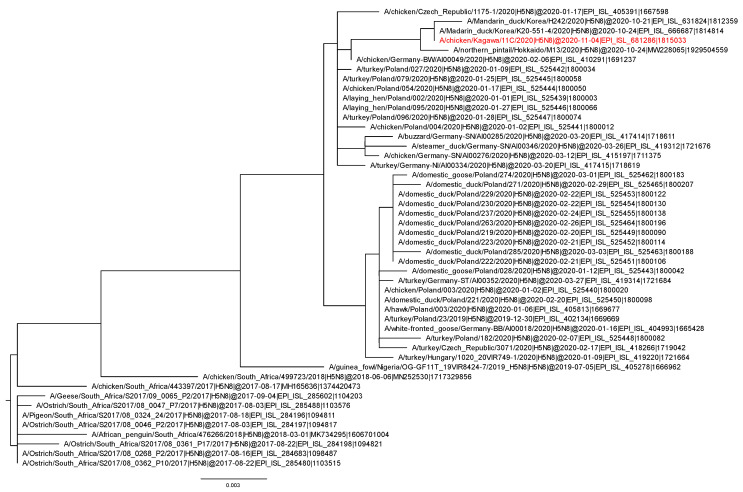
Expansion of clade 2.3.4.4B in phylogenetic tree of H5 subtype *HA* gene.

Phylogenetic analysis showed that the *HA* gene of Kagawa11C2020 belongs to clade 2.3.4.4B and clusters with those of A/northern pintail/Hokkaido/M13/2020 [4], A/mandarin duck/Korea/K20-551-4/2020 [20], and A/Mandarin duck/Korea/H242/2020 (GISAID isolate ID, EPI_ISL_631824) (Figure 1). The *HA* of Kagawa11C2020 also is closely related to those of H5N8 subtype European HPAIVs detected in wild-bird and poultry species during January–March 2020, such as A/chicken/Germany-BW/AI00049/2020. The phylogenetic trees of the other seven gene segments showed the same clustering pattern as that for the *HA* gene (data not shown). The branch including Kagawa11C2020 as well as the European strains stems from the HPAIVs isolated in South Africa in 2017. In contrast, the viruses in the Kagawa11C2020-containing branch are not closely related to the H5N1, H5N5, and H5N8 HPAIVs isolated from wild birds and poultry in Europe during September–November 2020, although they also belong to clade 2.3.4.4B.

Chickens were inoculated intranasally with Kagawa11C2020 to estimate the 50% chicken lethal dose (CLD_50_) and mean time to death (MTD). All of the chickens inoculated with 10^6^ EID_50_ and four of the five chickens inoculated with 10^5^ EID_50_ died, whereas all of the chickens inoculated with the 10^2^ or 10^4^ EID_50_ dose survived. All chickens that died from infection showed depression beginning on 3 dpi at the earliest (one chicken), but comb cyanosis occurred in only one chicken in each of the 10^5^ and10^6^ EID_50_ groups. The MTD of chickens inoculated with 10^6^ EID_50_ was 134.4 h, and the CLD_50_ of Kagawa11C2020 was 10^4.63^ (Table). None of the chickens that survived in 10^2^, 10^4^ and 10^5^ EID_50_ inoculation groups was ELISA-positive for antibody against type A influenza virus; they had therefore not been infected with the virus.

By comparing the survival of chickens, inoculated with 10^6^ EID_50_ of H5 HPAIVs isolated previously in Japan during 2004 through 2018 [5,11,18,21,22], showed that the survivability of Kagawa11C2020 was significantly higher than those of the other Japanese isolates except for A/chicken/Miyazaki/7/2014 (Miyazaki2014, H5N8), A/duck/Chiba/26-372-48/2014 (Chiba2014, H5N8) and A/Muscovy duck/Aomori/1-3T/2016 (Aomori2016, H5N6) (Table 1). Furthermore, the MTD was significantly longer in chickens infected with Kagawa11C2020 than in those infected with previous viruses except Miyazaki2014 (Table 1).

**Figure 2:**
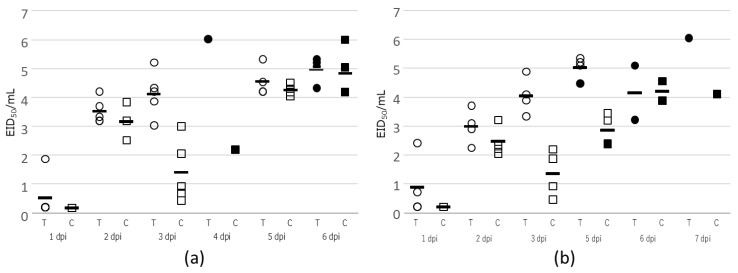
Kinetics of viral titer in tracheal (T) and cloacal (C) swabs collected from infected chickens. (**a**) 10^6^ EID_50_ inoculation group. (**b**) 10^5^ EID_50_ inoculation group. Circle, viral titer in tracheal swab; square, viral titer in cloacal swab; bar, the mean of viral titer; black circles and squares, viral titer in tracheal and cloacal swab, respectively, at time of death. The detection limit of viral titer was 10^0.2^ EID_50/mL_.

In our examination of virus shedding from intranasally inoculated chickens, virus was detected at 1 dpi in one of the five tracheal swabs from chickens inoculated with either 10^6^ EID_50_ or 10^5^ EID_50_ virus. All infected chickens in the 10^6^ EID_50_ and 10^5^ EID_50_ inoculation groups shed virus on 2 dpi (Figure 2). These results indicated that virus was shed from the trachea and in feces before clinical signs caused by the Kagawa11C2020 HPAIV became apparent. Therefore, chickens infected with Kagawa11C2020 might cause greater pollution of the farm environment before being noticed than birds infected with HPAIVs with shorter MTDs.

The maximum viral titer in tracheal and cloacal swabs collected from chickens experimentally infected with Kagawa11C2020 occurred on the day of death or 1 day before death (Figure 2). In addition, the mean maximum viral titers of tracheal and cloacal swabs from chickens experimentally inoculated with 10^6^ EID_50_ of Kagawa11C2020 were similar to, or significantly higher than, those from birds inoculated with other Japan isolates, A/Mandarin duck/Miyazaki/22M-765/2011 (MiyazakiMD2011, H5N8), A/chicken/Shimane/1/2010 (Shimane2010C, H5N1), Miyazaki2014 and A/northern goshawk/Tokyo/1301B003T/2018 (Tokyo2018, H5N6) (Table 1).

Infections of migratory wild birds, such as white-fronted goose, with those viruses in Europe that were prevalent during last winter from 2019 to 2020 were evident [23]. These species of migratory wild birds tend to winter in Europe and breed in Siberia during the summer, where they may co-mingle with migratory birds that winter in Asia. Therefore, migratory birds might have carried H5N8 HPAIVs that were isolated in early 2020 from Europe to Siberia, where the viruses could have been passed to birds that migrate to far-east Asia, thus providing a mechanism for the poultry outbreaks that occurred in Korea and Japan [12]. Although the intermediate ancestor of Kagawa11C2020 was not found in Siberia during summer 2020, intense surveillance for AIVs in Siberia during various birds’ breeding seasons is important, so that the poultry industry in this region might have advance warning of potential HPAI. To this end, it is important to identify where co-mingling of European and Asian migratory birds occurs.

## Figures and Tables

**Table 1:** Mean time to death, 50% chicken lethal dose (CLD_50_), results of survival analysis, and mean maximum viral titers in tracheal and cloacal swabs during the observation period of chickens experimentally inoculated with 10^6^ EID_50_ of H5 highly pathogenic avian influenza viruses isolated in Japan from 2004 through 2020.

Strain Name	Abbreviation	Subtype	Mean Time to Death (MTD, h)	Survival Analysis	log_10_CLD_50_	Mean Maximum Viral Titer during Observation Period (log_10_EID_50/mL_)		References
				(*p*)		Tracheal Swabs (*p*-Value)	Cloacal Swabs (*p*-Value)	
A/chicken/Kagawa/11C/2020	Kagawa11C2020	H5N8	134.4	N.A.	4.63	5.236	4.696	This study
A/chicken/Yamaguchi/7/2004	Yamaguchi2004	H5N1	48 *†	0.002 **	2.00 †	5.570 (0.499)	5.722 (0.146)	This study
A/chicken/Shimane/1/2010	Shimane2010	H5N1	58.8 *	0.003 **	3.00	5.561 (0.500)	3.119 *** (0.024)	[22]
A/chicken/Miyazaki/S4/2011	Miyazaki2011	H5N1	51.6 *	0.002 **	4.33	5.083 (0.500)	4.129 (0.481)	
A/Mandarin duck/Miyazaki/22M-765/2011	MiyazakiMD2011	H5N1	75.6 *	0.002 **	3.00	3.708 *** (0.017)	1.129 *** (0.000)	
A/chicken/Miyazaki/7/2014	Miyazaki2014	H5N8	120	0.314	4.50	5.199 (0.500)	2.607 *** (0.002)	[21]
A/duck/Chiba/26-372-48/2014	Chiba2014	H5N8	96	0.03	3.50	5.411 (0.500)	4.155 (0.491)	
A/chicken/Niigata/1-1T/2016	Niigata2016	H5N6	58.8 *	0.002 **	3.25	6.199 (0.199)	4.757 (0.500)	[18]
A/Muscovy duck/Aomori/1-3T/2016	Aomori2016	H5N6	70.5 *	0.284	5.25	6.041 (0.313)	3.729 (0.247)	
A/duck/Hyogo/1/2016	Hyogo2016	H5N6	57.6 *	0.002 **	5.00	5.480 (0.500)	4.732 (0.500)	
A/mute swan/Kyoto/1T/2016	Kyoto2016	H5N6	55.2 *	0.002 **	3.00	5.787 (0.472)	4.399 (0.500)	
A/chicken/Kumamoto/1-2C/2016	Kumamoto2016	H5N6	49.2 *	0.002 **	5.00	5.654 (0.497)	4.654 (0.500)	
A/chicken/Gifu/1-1T/2017	Gifu2017	H5N6	49.2 *	0.002 **	5.00	5.916 (0.405)	5.424 (0.410)	
A/chicken/Miyazaki/2-2C/2017	Miyazaki2017	H5N6	55.2 *	0.002 **	4.75	5.957 (0.376)	4.657 (0.500)	
A/chicken/Kagawa/1T-1/2018	Kagawa2018	H5N6	52 *	0.001 **	4.63	4.435 (0.290)	3.518 (0.106)	[11]
A/northern goshawk/Tokyo/1301B003T/2018	Tokyo2018	H5N6	57.6 *	0.002 **	4.38	3.591 *** (0.009)	3.763 (0.269)	
A/jungle crow/Hyogo/2803E023C/2018	Hyogo2018	H5N6	67.2 *	0.005 **	5.00	4.624 (0.446)	3.424 (0.090)	

*: Mean time to death was significantly shorter than for Kagawa11C2020. **: Significantly different from Kagawa11C2020 according to survival analysis. N.A represents “not applicable”. ***: Mean of maximum viral titer was significantly lower than for Kagawa11C2020. †: These values were retrieved from reference [5].

## Data Availability

The data that support the findings of this study are available from the corresponding author upon reasonable request.

## References

[1] Xu X., Subbarao K., Cox N.J., Guo Y. (1999). Genetic Characterization of the Pathogenic Influenza A/Goose/Guangdong/1/96 (H5N1) Virus: Similarity of Its Hemagglutinin Gene to Those of H5N1 Viruses from the 1997 Outbreaks in Hong Kong. Virology.

[2] Lycett S.J., Duchatel F., Digard P. (2019). A brief history of bird flu. Philos. Trans. R. Soc. B Biol. Sci..

[3] World Organization for Animal Health, January 2021 Update on Avian Influenza in Animals (Types H5 and H7). https://www.oie.int/en/animal-health-in-the-world/update-on-avian-influenza/2020.

[4] Isoda N., Twabela A.T., Bazarragchaa E., Ogasawara K., Hayashi H., Wang Z.-J., Kobayashi D., Watanabe Y., Saito K., Kida H. (2020). Re-Invasion of H5N8 High Pathogenicity Avian Influenza Virus Clade 2.3.4.4b in Hokkaido, Japan, 2020. Viruses.

[5] Nakamura K., Imada T., Imai K., Yamamoto Y., Tanimura N., Yamada M., Mase M., Tsukamoto K., Yamaguchi S. (2008). Pathology of Specific-Pathogen-Free Chickens Inoculated with H5N1 Avian Influenza Viruses Isolated in Japan in 2004. Avian Dis..

[6] WHO/OIE/FAO/H5N1 Evolution Working Group (2008). Toward a Unified Nomenclature System for Highly Pathogenic Avian Influenza Virus (H5N1). Emerg. Infect. Dis..

[7] Sakoda Y., Ito H., Uchida Y., Okamatsu M., Yamamoto N., Soda K., Nomura N., Kuribayashi S., Shichinohe S., Sunden Y. (2012). Reintroduction of H5N1 highly pathogenic avian influenza virus by migratory water birds, causing poultry outbreaks in the 2010–2011 winter season in Japan. J. Gen. Virol..

[8] Shivakoti S., Ito H., Otsuki K., Ito T. (2010). Characterization of H5N1 highly pathogenic avian influenza virus isolated from a mountain hawk eagle in Japan. J. Vet. Med. Sci..

[9] Uchida Y., Mase M., Yoneda K., Kimura A., Obara T., Kumagai S., Saito T., Yamamoto Y., Nakamura K., Tsukamoto K. (2008). Highly Pathogenic Avian Influenza Virus (H5N1) Isolated from Whooper Swans, Japan. Emerg. Infect. Dis..

[10] Kanehira K., Uchida Y., Takemae N., Hikono H., Tsunekuni R., Saito T. (2015). Characterization of an H5N8 influenza A virus isolated from chickens during an outbreak of severe avian influenza in Japan in April 2014. Arch. Virol..

[11] Mine J., Uchida Y., Nakayama M., Tanikawa T., Tsunekuni R., Sharshov K., Takemae N., Sobolev I., Shestpalov A., Saito T. (2019). Genetics and pathogenicity of H5N6 highly pathogenic avian influenza viruses isolated from wild birds and a chicken in Japan during winter 2017–2018. Virology.

[12] Saito T., Tanikawa T., Uchida Y., Takemae N., Kanehira K., Tsunekuni R. (2015). Intracontinental and intercontinental dissemination of Asian H5 highly pathogenic avian influenza virus (clade 2.3.4.4) in the winter of 2014–2015. Rev. Med. Virol..

[13] Takemae N., Tsunekuni R., Sharshov K., Tanikawa T., Uchida Y., Ito H., Soda K., Usui T., Sobolev I., Shestopalov A. (2017). Five distinct reassortants of H5N6 highly pathogenic avian influenza A viruses affected Japan during the winter of 2016–2017. Virology.

[14] Rozewicki J., Li S., Amada K.M., Standley D.M., Katoh K. (2019). MAFFT-DASH: Integrated protein sequence and structural alignment. Nucleic Acids Res..

[15] Price M.N., Dehal P.S., Arkin A.P. (2010). FastTree 2—Approximately Maximum-Likelihood Trees for Large Alignments. PLoS ONE.

[16] Ambaut A. (2009). Computer Program Distributed by the Author. http://tree.bio.ed.ac.uk/software/figtree/.

[17] World Organization for Animal Health (2018). Manual of Diagnostic Tests and Vaccines for Terrestrial Animals 2019. Chapter 3.3.4. https://www.oie.int/fileadmin/Home/eng/Health_standards/tahm/3.03.04_AI.pdf.

[18] Uchida Y., Mine J., Takemae N., Tanikawa T., Tsunekuni R., Saito T. (2019). Comparative pathogenicity of H5N6 subtype highly pathogenic avian influenza viruses in chicken, Pekin duck and Muscovy duck. Transbound. Emerg. Dis..

[19] Swayne D.E. Animal Influenza, 2016; p. 208. https://onlinelibrary.wiley.com/doi/pdf/10.1002/9781118924341.

[20] Jeong S., Lee D.-H., Kwon J.-H., Kim Y.-J., Lee S.-H., Cho A.Y., Kim T.-H., Park J.-E., Lee S.-I., Song C.-S. (2020). Highly Pathogenic Avian Influenza Clade 2.3.4.4b Subtype H5N8 Virus Isolated from Mandarin Duck in South Korea, 2020. Viruses.

[21] Tanikawa T., Kanehira K., Tsunekuni R., Uchida Y., Takemae N., Saito T. (2016). Pathogenicity of H5N8 highly pathogenic avian influenza viruses isolated from a wild bird fecal specimen and a chicken in Japan in 2014. Microbiol. Immunol..

[22] Uchida Y., Suzuki Y., Shirakura M., Kawaguchi A., Nobusawa E., Tanikawa T., Hikono H., Takemae N., Mase M., Kanehira K. (2012). Genetics and infectivity of H5N1 highly pathogenic avian influenza viruses isolated from chickens and wild birds in Japan during 2010–11. Virus Res..

[23] King J., Schulze C., Engelhardt A., Hlinak A., Lennermann S.-L., Rigbers K., Skuballa J., Staubach C., Mettenleiter T.C., Harder T. (2020). Novel HPAIV H5N8 Reassortant (Clade 2.3.4.4b) Detected in Germany. Viruses.

